# NT-ProBNP Predicts Total Mortality, Emergency Department Visits, Hospitalization, Intensive-Care Unit Admission, and Cardiovascular Events in Hemodialysis Patients

**DOI:** 10.3390/jcm8020238

**Published:** 2019-02-12

**Authors:** Yi-Hsin Chen, Yun-Ching Fu, Ming-Ju Wu

**Affiliations:** 1 Institute of Clinical Medicine, National Yang-Ming University, Taipei 112, Taiwan; nephp06@gmail.com (Y.-H.C.); yunchingfu@gmail.com (Y.-C.F.); 2 Department of Nephrology, Taichung Tzu Chi Hospital, Buddhist Tzu Chi Medical Foundation, Taichung 427, Taiwan; 3 School of Medicine, Tzu Chi University, Hualien 907, Taiwan; 4 Section of Pediatric Cardiology, Department of Pediatrics, Taichung Veterans General Hospital, Taichung 407, Taiwan; 5 School of Medicine, Chung Shan Medical University, Taichung 402, Taiwan; 6 School of Medicine, College of Medicine, China Medical University, Taichung 404, Taiwan; 7 Division of Nephrology, Department of Internal Medicine, Taichung Veterans General Hospital, Taichung 407, Taiwan; 8 Rong-Hsing Research Center for Translational Medicine and Graduate Institute of Biomedical Sciences, College of Life Science, National Chung Hsing University, Taichung 402, Taiwan

**Keywords:** NT-proBNP, time-varying, clinical outcomes, hemodialysis, area under the curve (AUC)

## Abstract

N-terminal pro b-type natriuretic peptide (NT-proBNP) was considered a prognostic factor for mortality in hemodialysis patients in previous studies. However, NT-proBNP has not been fully explored in terms of predicting other clinical outcomes in hemodialysis patients. This study aimed to investigate if NT-proBNP could predict emergency department (ED) visits, hospitalization, admission to intensive-care unit (ICU), and cardiovascular incidents in hemodialysis patients. Serum NT-proBNP and other indicators were collected in 232 hemodialysis patients. Patients were followed up for three years or until mortality. Outcomes included mortality, number of ED visits, hospitalizations, admissions to ICU, and cardiovascular events. NT-proBNP was found to predict recurrent ER visits, hospitalization, admission to ICU, cardiovascular events, and mortality, after adjusting for covariates. Time-dependent area under the curve (AUC) was used to evaluate the NT-proBNP predicting ability. Using time-dependent AUC, NT-proBNP has good predictive ability for mortality, ED visit, hospitalization, ICU admission, and cardiovascular events with the best predictive ability occurring at approximately 1 year, and 5th, 62nd, 63rd, and 63rd days respectively. AUC values for predicting mortality, hospitalization, and ICU admission decreased significantly after one year. NT-proBNP can be applied in predicting ED visits but is only suitable for the short-term. NT-proBNP may be used for predicting mortality in the long term.

## 1. Introduction

N-terminal pro b-type natriuretic peptide (NT-proBNP) has been widely used as a prognostic indicator in heart failure patients [1]. NT-proBNP is secreted in ventricles when an elevation in blood pressure is detected. This biomarker will also increase when patients are in decompensated heart failure [2]. Previous studies have indicated NT-proBNP reduction percentage can predict total mortality, cardiovascular mortality, re-hospitalization, and cardiac events [3,4,5]. 

Cardiovascular events are common in hemodialysis patients. Mortality prediction by using NT-proBNP has been previously investigated [6,7]. However, few studies have investigated the role of NT-proBNP in predicting other clinical endpoints. Another issue is the higher cut-point of NT-proBNP when predicting mortality in hemodialysis patients when compared to general heart failure patients [8,9]. Svensson’s study indicated hemodialysis patients with NT-proBNP more than 12,200 pg/mL had a 3 times higher risk of death than patients below the cut-off value hazard ratio (HR) 3.05 95% confidence interval (CI) 1.96–4.77, *p* < 0.0001) [9]. Madsen’s study found predialysis NT-proBNP with a cutoff value 4079 pg/mL predicted subsequent death (area under the curve: 0.718) [8]. The prognostic impact of NT-proBNP in predicting mortality in hemodialysis patients is established but it is unknown if the same applies to predicting emergency department (ED) visits, hospitalization, and intensive-care unit (ICU) admission [8,9].

Gutiérrez et al. found that serial NT-proBNP can predict mortality in hemodialysis patients after controlling baseline biochemistry values [10]. However, there is still a lack of studies evaluating the time-dependent prediction ability of NT-proBNP. Traditional receiver operating characteristic (ROC) curve analysis was used as fixed over time when evaluating a range of trade-offs achieved by a diagnostic test. However, many disease outcomes are time dependent. Both the disease status and marker value are always changed in follow up studies [11,12]. Patients who are disease-free earlier may develop the disease later due to longer study follow-up, and also their marker value as a diagnostic test may change over time. A receiver operating characteristic (ROC) curve as a function of time therefore is more appropriate [12,13]. In this study, we aimed to determine the predictive ability of serial NT-proBNP values through time-dependent ROC curve on different time points. Time-dependent ROC of the aforementioned clinical endpoints was also determined in this study. We hypothesized that NT-proBNP values would show good prognostic value for the investigated outcomes.

## 2. Experimental Section

### 2.1. Ethics

All subjects gave their informed consent for inclusion before they participated in the study. The study was conducted in accordance with the Declaration of Helsinki. This study was approved by Institutional Review Board at Taichung Tzuchi Hospital (IRB100-106).

### 2.2. Patients

A total of 232 hemodialysis patients were recruited in the dialysis center at Taichung Tzuchi Hospital in this prospective survey and followed-up for three years. The following inclusion criteria were applied: at least 18 years old, receiving hemodialysis three times per week, with each session lasting for 4  h. The following exclusion criteria were applied: malignancies, acute infections, autoimmune diseases, and active drug abuse. 

### 2.3. Outcome Events Definition

We reviewed patients’ electronic medical records (EMR) from index date (first serum NT-proBNP measurement) until death with any cause, outmigration, or followed them for three years to capture all ED visits, hospitalizations, ICU admissions, cardiovascular events. We captured the number and date of ED visits with any cause. Time and counts of admission to ICU were also analyzed. Cardiovascular events were also further analyzed from our medical records including acute myocardial infarction, stroke, coronary heart disease according to previous study [14]. We identified cardiovascular events in records by diagnoses aforementioned and verified them with International Classification of Diseases, Clinical Modification, Ninth Revision (ICD-9) code including myocardial infarction (410.x, 411.x, 412.x, 413.x, 414.x, 429.2, and v45.81), stroke (433.11, 433.91, 434.91, 435.x, 436.x, 437.9x, 438.x), coronary artery bypass graft surgery (ICD-9-Procedure 36.1x), or percutaneous coronary intervention (ICD-9-Procedure 36.06, 36.07, 0.66) [15]. 

### 2.4. Comorbidities of Interest

We reviewed patients’ EMR and identified the following diagnoses as comorbidities: hypertension, hyperlipidemia, coronary artery disease, and diabetes. We also used prescription records, laboratory values, and vital signs data to identify these comorbidities in order to minimize potential underdiagnosis and undercoding. Specifically, individuals were classified as having hypertension if they had 2 or more blood pressure readings on different days in which their diastolic blood pressure reading was over 80, if their systolic blood pressure was over 130 during the study period, or if they received any antihypertensive medications [16,17,18]. Individuals were classified as having hyperlipidemia if they had 2 or more readings on different days in which their total cholesterol levels were over 200, low-density lipoprotein cholesterol levels were over 130, triglyceride levels were over 150, or high density lipoprotein cholesterol levels were under 60; or if they received any lipid-lowering medications. Coronary artery disease was defined as any evidence of coronary atherosclerotic plaque (obstructive or nonobstructive) on coronary angiography, ischemia on non-invasive cardiac testing, or past history of myocardial infarction/percutaneous coronary intervention/surgical revascularization. We defined patients as having diabetes based on a diagnosis of diabetes in medical records or as having receiving prescribed or redeemed blood glucose–lowering drugs. 

### 2.5. Procedures

All patient’s clinical or demographic characteristics were reviewed and collected. Blood testing was performed via venous sampling after overnight fasting. The plasma was separated and frozen at negative 70 degrees Celsius for further analysis. Venous sample was collected in the middle of the week before hemodialysis. The average follow-up time was 2.73 years.

### 2.6. Serum Analyses

Serum NT-proBNP was measured by enzyme-linked fluorescent assay (VIDAS NT-proBNP, bioMérieux, France) [19]. All other clinical biochemical parameters were measured by Siemens Dimension RxL system [20]. 

### 2.7. Statistical Analysis 

Non-normally distributed data were presented as median (interquartile (25th–75th percentiles) range), and normally distributed variables as mean ± SD. A Shapiro–Wilk test was performed to check normality of variables. Continuous variables normally distributed for each baseline NT-proBNP quartile were compared with analysis of variance (ANOVA) followed by the Bonferroni post hoc test. Categoric variables for each quartile were compared by chi-square test followed by Bonferroni test in multiple comparisons. Continuous variables not normally distributed compared with the Kruskal–Wallis test. Continuous variables tested for trend in each quartile were performed with linear trend test if variables were normally distributed, but with the Jonckheere–Terpstra test if variables were not normally distributed. Categorical variables tested for trend were performed with linear-by-linear trend test.

A Kaplan–Meier curve was used to summarize the survival distribution for all-cause mortality. A non-parametric, recurrent event survival curve was used to summarize the survival distribution for hospitalization, ED visits, ICU admission, and cardiovascular events [21]. More than three observation points were obtained from each patient. Recurrent event models were used to determine the relative hazard of recurrent events between NT-proBNP quartiles.

NT-proBNP values were logarithmically transformed before entering regression analysis for non-normally distributed data and are represented as lnBNP. The Andersen–Gill extension of the Cox proportional hazards model was used for multiple events per hemodialysis patient and in association with lnBNP as a time-varying covariable [22]. Covariates were selected by backward selection of models performed with the function stepAIC in the package MASS [23]. This approach removes covariates with the smallest contribution to model likelihood while penalising redundancy. The selection procedure terminated with a final model when removing any single covariate did not improve Akaike information criterion.

For the predictive ability of clinical outcomes at different time points, time-dependent receiver operating characteristic (survival ROC package, R language) was applied to display and compare the sensitivity and specificity of the predictive models based on the survival analysis [24]. A time-dependent area under the curve (AUC (t)) was also plotted to investigate the predictive accuracy on different time points. All *p*-values were two-tailed. A *p*-value of less than 0.05 was considered statistically significant. The SAS software Version 9.4 (SAS Institute Inc., Cary, NC, USA) and R software Version 2.6.1 (R Foundation for Statistical Computing, Vienna, Austria) with survival package were used. 

## 3. Results

### 3.1. Patient Characteristics 

Patients were grouped into quartiles according to their baseline NT-proBNP value. The percentages of coronary artery disease in Q3 and Q4 were higher than Q1 and Q2 (*p* = 0.005, Q1: 51.7%, Q2: 36.2%, Q3: 69%, Q4: 67.2%) (Table 1). The trend test for percentages of coronary artery disease showed an increasing trend with increasing NT-proBNP quartiles (P-trend = 007). The proportion of patients in NYHA class III or IV was significantly greater in the Q3 and Q4 (45.7%, 26.1%) than in the Q1 and Q2 (15.2%, 13.0%) (*p* = 0.002). The trend test demonstrated the higher proportion of severe heart failure in increasing NT-proBNP quartile (P-trend = 0.001). Proportion of male participants was not significantly different between quartiles (*p* = 0.064), but revealed a linear trend with increasing quartiles with an increasing proportion of female participants (P-trend = 0.022). 

**Figure 1:**
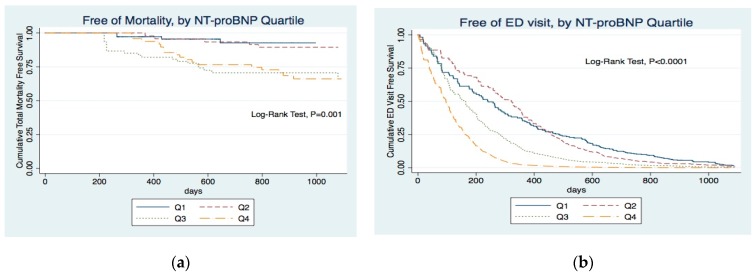

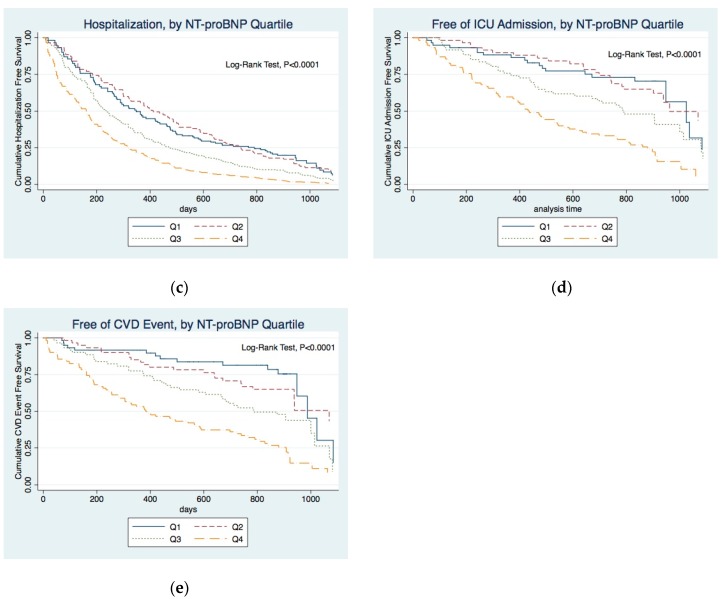
Kaplan–Meier curve for five outcome events (**a**) Kaplan–Meier curve for mortality according to quartiles of NT-proBNP levels at study entry. (**b**) Probability of emergency department (ED) visit for all participants according to quartiles of NT-proBNP levels. (**c**) Probability of hospitalization for all participants according to quartiles of NT-proBNP levels. (**d**) Probability of intensive-care unit (ICU) admission for all participants according to quartiles of NT-proBNP levels. (**e**) Probability of cardiovascular (CVD) event for all participants according to quartiles of NT-proBNP levels.

Kaplan–Meier estimates of 3-year mortality were significantly worse with increasing NT-proBNP quartiles of (Figure 1a; log-rank test, *p* = 0.001). Recurrent ED visit, hospitalization, admission to ICU, and cardiovascular event were also compared among the four quartiles and revealed the same trend (Figure 1b–e; log-rank test, *p* < 0.0001). Number of each outcome event by NT-proBNP quartile was also listed in Table 2. Increasing numbers in each outcome was demonstrated with increasing NT-proBNP quartiles.

Andersen–Gill extension of the Cox proportional hazards model was used for revealing the effect of lnBNP on clinical outcomes. Age, serum C-reactive protein (CRP), albumin (ALB), were selected as covariates by backward selection by function stepAIC for model of total mortality. Mortality risk increased 1.54 for per unit of lnBNP increment (Table 3, adjusted HR 1.54, CI: 1.08–2.19, *p* = 0.0158). Covariates selected by backward selection for model of ED visit included Age, systolic blood pressure (SBP), diastolic blood pressure (DBP), body mass index (BMI), ALB, alanine aminotransferase (ALT), uric acid (UA), serum sodium (Na), serum potassium (K), serum phosphorus (P), hemoglobin (Hb), parathyroid hormone (PTH), ferritin, hypertension (HTN), coronary artery disease (CAD). Risk of ED visit increased 1.36 for per unit of lnBNP increment (Table 3, adjusted HR 1.36, CI: 1.24–1.50, *p* < 0.0001). Covariates of adjusted model of hospitalization included SBP, ALT, UA, K, P, ferritin, diabetes, ALB, CRP. Risk of hospitalization increased 1.35 for per unit of lnBNP increment (Table 3, adjusted HR 1.35, CI: 1.19–1.52, *p* < 0.0001). Covariates selected for model of ICU admission included SBP, blood urea nitrogen, P, ferritin, diabetes, CAD, ALB, CRP. Risk of ICU admission increased 1.49 for per unit of lnBNP increment (adjusted HR 1.49, CI: 1.20–1.85, *p* = 0.0003). Covariates after backward selection in model of cardiovascular event included Age, SBP, UA, Hb, diabetes, CAD, CRP. Risk of cardiovascular event increased 1.67 for per unit of lnBNP increment (adjusted HR 1.67, CI: 1.33–2.10, *p* < 0.0001).

### 3.2. Predictive Ability of N-Terminal Pro B-Type Natriuretic Peptide (NT-ProBNP) Values for Different Clinical Outcomes at Different Time Points 

#### 3.2.1. Mortality 

**Figure 2:**
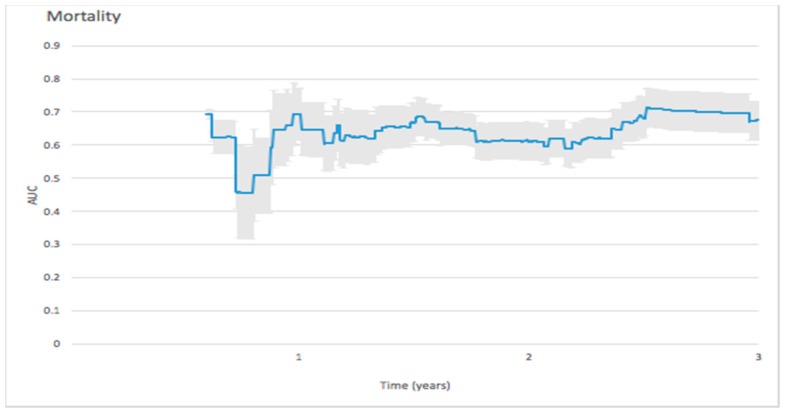
Forecast time-dependence of the area under the curve (AUC) for NT-proBNP-based prediction of mortality among hemodialysis patients, with the corresponding 95% confidence bands.

The AUC value reached 0.6926 on the 267th day, gradually decreased, reached 0.69 after the first year, then reached its highest value of 0.713 at 2.5 years. (Figure 2)

#### 3.2.2. Emergency Department (ED) Visit

**Figure 3:**
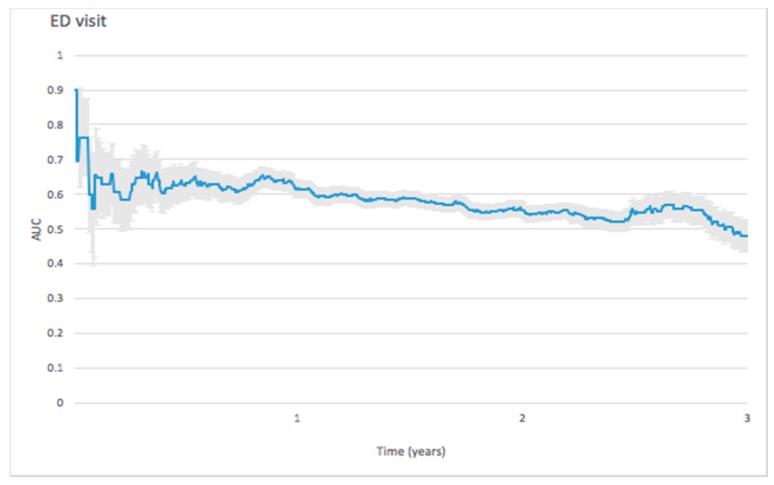
Forecast time dependence of the AUC for NT-proBNP-based prediction of emergency department (ED) visit among hemodialysis patients, with the corresponding 95% confidence bands.

The initial peak of AUC value for predicting ED visit reached 0.8987 in the 5th day, then dropped to 0.5575 in the 37th day, and continued to decrease during the 3-year follow-up. (Figure 3).

#### 3.2.3. Hospitalization

**Figure 4:**
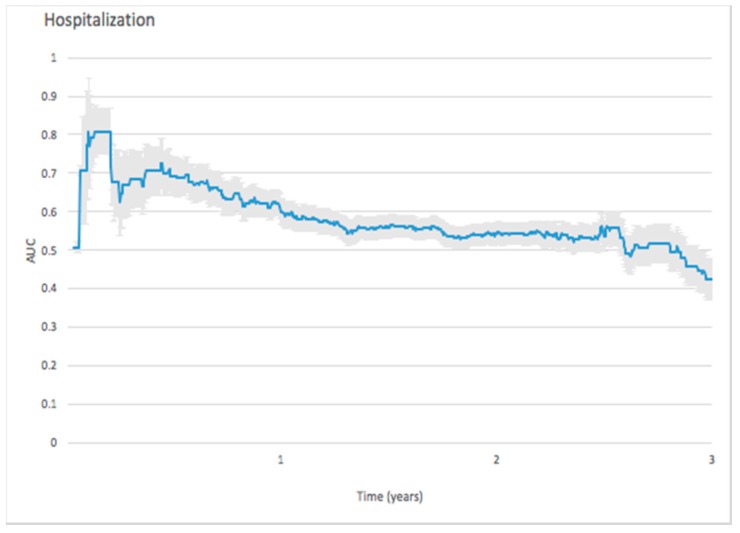
Forecast time dependence of the AUC for NT-proBNP-based prediction of hospitalization among hemodialysis patients, with the corresponding 95% confidence bands.

On predicting hospitalization, the initial peak of AUC value reached 0.8088 in the 50th day and then gradually dropped to 0.6 after one year. (Figure 4)

#### 3.2.4. Intensive-Care Unit (ICU) Admission 

**Figure 5:**
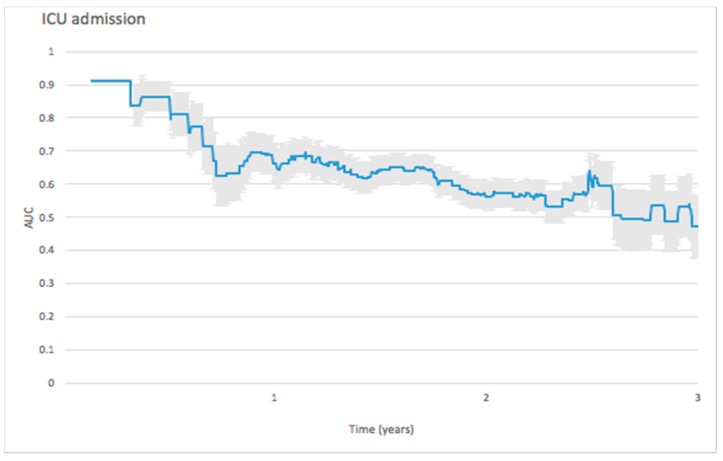
Forecast time dependence of the AUC for NT-proBNP-based prediction of ICU admission among hemodialysis patients, with the corresponding 95% confidence bands.

The ICU admission predictive ability, as measured by the AUC value, reached a peak of 0.9111 on the 62nd day, gradually decreasing thereafter to 0.6 after the 640th day. (Figure 5)

#### 3.2.5. Cardiovascular Events

**Figure 6:**
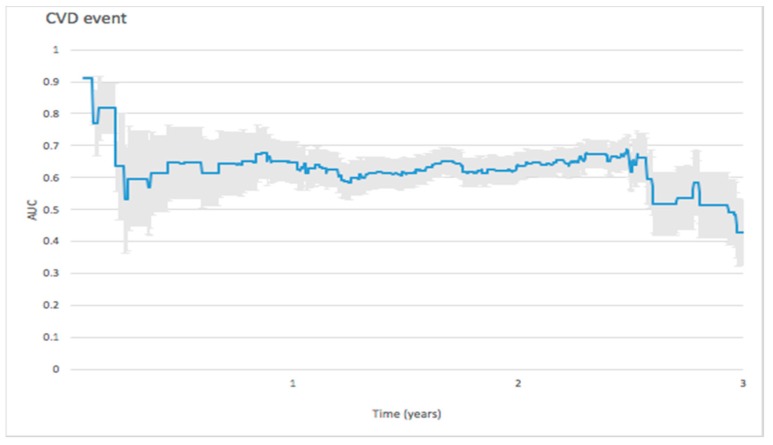
Forecast time dependence of the AUC for NT-proBNP-based prediction of cardiovascular (CVD) event among hemodialysis patients, with the corresponding 95% confidence bands.

For predicting cardiovascular events, the initial peak AUC value reached 0.9116 on the 25th day and dropped rapidly to 0.7698 on the 41st day. The AUC value remained stable around 0.6 thereafter (Figure 6).

## 4. Discussion

We aimed to investigate the predictive ability of NT-proBNP values in hemodialysis patients regarding different clinical outcomes. We found NT-proBNP can predict ED visit, hospitalization, ICU admission, cardiovascular event, and mortality after controlling for other covariates. Prediction ability of NT-proBNP assessed by time-dependent ROCs revealed that predicting ED visit is most accurate within a one-week time-frame, predicting cardiovascular events is suitable within one month, and predicting ICU admission and hospitalization is most accurate within two months. Predicting mortality is most accurate within one year, with an AUC of around 0.69. 

NT-proBNP is also used to reflect glomerular filtration rate decrements in chronic kidney disease patients [25], and the status of extracellular volume in addition to the magnitude of heart failure [26,27]. NT-proBNP values were higher in our patients than in the heart failure patients of previous studies. Patients with higher NT-proBNP had higher percentage of coronary artery disease in our study (Q1: 51.7%, Q2: 36.2%, Q3: 69%, Q4: 67.2%, *p* = 0.005). The proportion of diabetes was not different among the four quartiles (*p* = 0.39). Gutiérrez et al. found that higher NT-proBNP values were associated with increased rates of coronary artery disease in end-stage renal disease patients, which is in line with our study [10]. A higher proportion of female participants was observed with higher NT-proBNP in our study (*p* = 0.064, P-trend = 0.022). Previous study also demonstrated gender have a significant influence on BNP levels [28]. They found females had a higher BNP value than males in the general population. The clinical significance of this finding deserved further investigation in the future for hemodialysis patients.

In our survival analysis (Figure 1), higher NT-proBNP was associated with higher risk for five clinical outcomes. A study by Paniagua et al. showed NT-proBNP can independently predict mortality even after controlling for other clinical factors [6]. Our study also revealed that our patients with higher NT-proBNP had lower survival probability (Figure 1a). NT-proBNP remained an independent predictive factor of mortality even in multivariate Cox analysis (Table 3, adjusted HR: 1.51, *p* = 0.031). NT-proBNP is used in the differential diagnosis of pulmonary and cardiac dyspnea among patients in ED [29,30]. However, there are no previous studies about whether NT-proBNP can predict ED visit in hemodialysis patients. Our study revealed hemodialysis patients with higher NT-proBNP are more likely to visit the ED. (Figure 1b) NT-proBNP was positively associated with ED visits in the multivariate Cox model (adjusted HR 1.35; 95% CI 1.15–1.58; *p* = 0.0001).

### 4.1. Hospitalization, ICU Admission, and Cardiovascular Events

NT-proBNP also provided valuable information to predict re-hospitalization associated with heart failure before discharge [31]. Verdiani et al. found higher NT-ProBNP values during hospital stay predict readmission [5]. Our data revealed that an increment of one unit in the NT-proBNP value will increase the chances of all-cause hospitalization 1.32-fold. Stolz et al. found BNP levels were significantly higher in patients requiring ICU treatment in a cohort of 208 chronic obstructive pulmonary disease patients older than 40 years [32]. Our study indicates hemodialysis patients with higher NT-proBNP will have increased risk of ICU admission after controlling for other factors in multivariate model (adjusted HR 1.46; 95% CI 1.13–1.88; *p* = 0.003). NT-proBNP values were able to predict the risk of cardiovascular events (HR 1.65; 95% CI 1.29–2.11; *p* < 0.0001). These results are in line with Tsuchida et al.’s findings which indicated BNP levels predicted the risk of cardiovascular events [33]. 

### 4.2. Predictive Ability Over Time

#### 4.2.1. Mortality 

In the Masson et al. study, the AUC of NT-proBNP predicting 2-year all-cause mortality was 0.679 [34], which is similar to the values of 0.690 and 0.710 found in our study for 2- and 3-year all-cause mortality, respectively. The predictive ability for 3-year mortality was relatively stable. In patients with dyspnea at admission to ED [35], Januzzi et al. found that the AUC of predicting one-year mortality was 0.76. However, their study was based on dyspnea patients with heterogenous characteristics.

#### 4.2.2. ED Visit

The ED visit predictive ability revealed the highest AUC of 0.808 at the 5th follow-up day and AUC dropped to 0.557 at one month. No known previous studies were performed to reveal the role of NT-proBNP in predicting ED; most studies focus on the role of NT-proBNP value at ED admission in differentiating dyspnea patients [36,37].

#### 4.2.3. Predicting Hospitalization

The predictive ability of NT-proBNP remains controversial in some respects. Noveanu et al. used NT-proBNP to predict one-year hospitalization, but the AUC of NT-proBNP at presentation was only 0.49 (AUC: 0.49, CI: 0.34–0.63) [38]. Our study revealed that NT-proBNP can predict hospitalization up to 50 days (AUC: 0.81, CI: 0.75–0.87). The ability to predict hospitalization dropped to 0.6 after one year. Our findings are also compatible with the poor predictive ability found by Noveanu et al. [38], in an evaluation made at the time of hospitalization. Using NT-proBNP to predict hospitalization in our study demonstrated the best predictive ability occurring at approximately the 50th day, which can be used to make a prognosis of the number of beds necessary at the department. High levels of BNP are also reported to be related to 1- and 6-month rehospitalization and mortality in patients with decompensated heart failure [39,40,41]. 

#### 4.2.4. Predicting ICU Admission

For the prediction of ICU admission, Krzych et al. revealed that preoperative assessment of NT-proBNP levels in coronary artery bypass graft patients could be a valuable diagnostic method for predicting several postoperative complications, especially pulmonary outcomes and the requirement for hemodynamic support; it was also correlated with the length of ICU stay. BNP level >190 pg/mL was a predictor of an ICU stay lasting >5 days [42]. Our study showed that NT-proBNP could predict ICU admission up to the 62nd day with an AUC of 0.9111 which gradually decreased thereafter. 

#### 4.2.5. Cardiovascular Events

Wolsk et al. noted that in patients with a recent acute coronary syndrome and type 2 diabetes mellitus, BNP significantly improved C-statistics when added to risk models for each outcome examined and for cardiovascular death in a two-year follow-up study [43]. The predictive ability of BNP combined with other risk factors to predict the incidence of cardioembolic stroke was excellent in in a general population in an 8-year follow-up was verified by Nakamura et al. [44]. NT-proBNP could predict cardiovascular events on the 25th day with a peak value of AUC reaching 0.9116, dropping rapidly to 0.7698 on the 41st day.

### 4.3. Limitations

We did not include intradialytic weight gain and ultrafiltration volume in our analysis. Causes of emergency room visits were not collected in our study. Mortality was not treated as a competing risk in our model. To learn more about how uremia per se affects the predictive ability of NT-proBNP, a similar study design including patients with or without uremic changes would have been optimal. The follow-up time was limited to 3 years in our study; therefore, any long-term predictive ability was not determined. Extrapolating conclusions from the present study to patients without dialysis should be done cautiously.

## 5. Conclusions

In hemodialysis patients, NT-proBNP is an important predictor of clinical outcomes including mortality, ED visit, hospitalization, ICU visit, and cardiovascular events. However, predicting ED visit is only suitable in the short-term. Mortality prediction was accurate within one year. Predicting hospitalization, ICU admission, and cardiovascular event was more accurate within 2 months. Further studies on the long-term utility of NT-proBNP in dialysis patients are needed. 

## Figures and Tables

**Table 1:** Patient characteristic stratified by N-terminal pro b-type natriuretic peptide (NT-proBNP) quartile.

	NT-ProBNP Quartile					
Parameter	Q1 (<2296, *n* = 58)	Q2 (2296–4568, *n* = 58)	Q3 (4568–13,360, *n* = 58)	Q4 (>13,360, *n* = 58)	*p*	P-trend
Age, mean (SD), years	63.1 (13.0)	63.9 (12.7)	66.6 (12.6)	66.1 (12.6)	0.375	0.115
Gender, male (%)	40 (69)	29 (50)	29 (50)	27 (46.6)	0.064	0.022
SBP, mean (SD)	142.03 (19.945)	154.69 (49.49)	151.64 (28.01)	144.86 (24.65)	0.134	0.355
DBP, mean (SD)	76.1 (12.78)	75.74 (14.82)	77.97 (14.45)	76.88 (14.99)	0.843	0.588
BMI, mean (SD)	24.54 (3.19)	24.21 (4.068)	24.53 (3.60)	23.19 (2.98)	0.125	0.070
ALT, median (IQR)	15 (12–21.25)	13.5 (10–20.5)	16.5 (11–21.25)	14.5 (11–22.5)	0.679	0.924
BUN, mean (SD)	84.98 (22.62)	81.74 (20.15)	81.17 (20.96)	80.81 (20.59)	0.700	0.292
UA, median (IQR)	8.25 (7.175–9.025)	7.85 (6.8–8.475)	7.65 (6.7–8.5)	7.7 (6.3–8.7)	0.238	0.053
Na, median (IQR)	135 (132–137)	134 (132–136)	135 (133–137)	134.5 (132–136.2)	0.491	0.961
K, median (IQR)	4.7 (4.175–5)	4.9 (4.375–5.225)	4.8 (4.375–5.4)	5 (4.25–5.425)	0.246	0.128
Ca, median (IQR)	8.7 (8.28–9.29)	8.68 (8.12–9.24)	8.42 (8.16–9.16)	8.72 (8.04–9.29)	0.855	0.543
P, median (IQR)	5.1 (4.375–5.8)	5.05 (4.1–5.9)	5.2 (4.5–6.525)	5.2 (4.175–6.5)	0.627	0.441
Hb, median (IQR)	10.6 (9.475–11.15)	9.9 (9.475–10.625)	10.2 (9.6–10.825)	10.3 (8.8–11.125)	0.427	0.537
PTH, median (IQR)	215.3 (74.3–422.1)	207.1 (73.2–355.2)	179.4 (86.4–275.2)	206.9 (69.5–455.6)	0.427	0.986
Ferritin, median (IQR)	346.9 (164–478.8)	378.3 (217–536.2)	299.4 (189.6–498.2)	505.3 (221.9–631.8)	0.072	0.055
HTN, *n* (%)	50 (86.2)	53 (91.4)	54 (93.1)	56 (96.6)	0.271	0.048
CAD, *n* (%)	30 (51.7)	21 (36.2)	40 (69) †	39 (67.2) †	0.005	0.007
DM, *n* (%)	41 (70.7)	32 (55.2)	37 (63.8)	40 (69)	0.399	0.934
Hyperlipidemia, *n* (%)	12 (20.7)	7 (12.1)	9 (15.5)	8 (13.8)	0.573	0.412
ALB, median (IQR)	4 (3.8–4.2)	4 (3.7–4.2)	3.9 (3.6–4.1)	4 (3.575–4.2)	0.385	0.22
CRP, median (IQR)	0.28 (0.12–0.69)	0.25 (0.11–0.99)	0.32 (0.13–0.93)	0.36 (0.13–0.94)	0.873	0.492
NYHA class III or IV, *n* (%)	7 (15.2%)	6 (10.3%)	12 (20.7%)	21 (36.2%) *†	0.002	0.001
Dialysis vintage years, median (IQR)	1.30 (0.31–3.35)	1.29 (0.33–4.12)	1.59 (0.25–4.31)	1.58 (0.33–3.16)	0.952	0.749

SD, standard deviation for variables with normal distribution; IQR, interquartile range for variable not normally distributed; SBP, systolic blood pressure; DBP, diastolic blood pressure; BMI, body mass index; ALT, alanine aminotransferase; UA, uric acid; Na, serum sodium; K, serum potassium; Ca, serum calcium; P, serum phosphorus; Hb, hemoglobin; PTH, serum parathyroid hormone; HTN, hypertension; CAD, coronary artery disease; DM, diabetes mellitus; ALB, serum albumin; CRP, C-reactive protein; NT-proBNP, N-terminal prohormone B-type natriuretic peptide, NHYA (New York Heart Association); P-trend, test for trend in each quartile, Jonckheere-Terpstra trend test was used for variables not normally distributed, linear trend test for variables with normally distributed, linear-by-linear trend test for categorical variables; continuous variables normally distributed for each quartile were compared with analysis of variance (ANOVA), categoric variables for each quartile were compared by chi-square test followed by Bonferroni test in multiple comparisons, Continuous variables not normally distributed compared with Kruskal-Wallis test, All *p* values reported are for two-tailed test, and a two-tailed alpha of <0.05 was considered statistically significant; * indicates *p* less than 0.05 versus Q1, † versus Q2.

**Table 2:** Number of each outcome event by NT-proBNP quartile.

NT-proBNP Quartile				
Events Observed	Q1 (<2296)	Q2 (2296–4568)	Q3 (4568–13,360)	Q4 (>13,360)
Mortality	3	7	14	22
ED visit	157	222	303	400
Hospitalization, *n* (%)	39 (67)	42 (72)	46 (79)	50 (86)
ICU admission	23	33	45	65
CVD event	18	30	39	67

ED, emergency department; ICU, admission to intensive-care unit; CVD, cardiovascular event; Hospitalization n (%), number of individuals who had at least 1 hospitalization and percentage.

**Table 3:** Hazard rate of clinical outcomes for lnBNP analysis.

Outcome	Unadjusted Model		Adjusted Model	
	HR (95% CI)	*p*-Value	HR (95% CI)	*p*-Value
Total mortality	1.94 (1.39, 2.71)	<0.0001	1.54 (1.08, 2.19)	0.0158
ED visit	1.44 (1.23, 1.68)	<0.0001	1.36 (1.24, 1.50)	<0.0001
Hospitalization	1.42 (1.21, 1.65)	<0.0001	1.35 (1.19, 1.52)	<0.0001
ICU admission	1.65 (1.30, 2.11)	<0.0001	1.49 (1.20, 1.85)	0.0003
CVD	1.71 (1.33, 2.17)	<0.0001	1.67 (1.33, 2.10)	<0.0001

HR, hazard ratio; lnBNP, NT-proBNP is logarithmically transformed; ED, emergency department; ICU, admission to intensive-care unit; CVD, cardiovascular event; Andersen-Gill extension of the Cox proportional hazard model was used here; Covariates for adjustment in model of mortality included age, serum C-reactive protein (CRP), albumin (ALB); covariates for model of ED included age, systolic blood pressure (SBP), diastolic blood pressure (DBP), body mass index (BMI), ALB, alanine aminotransferase (ALT), uric acid (UA), serum sodium, serum potassium(K), serum phosphorus (P), hemoglobin (Hb), parathyroid hormone (PTH), ferritin, hypertension (HTN), coronary artery disease (CAD); covariates for model of hospitalization included SBP, ALT, UA, K, P, ferritin, diabetes, ALB, CRP; covariates for model of ICU admission included SBP, blood urea nitrogen, P, ferritin, diabetes, CAD, ALB, CRP; covariates for model of CVD event included age, SBP, UA, Hb, diabetes, CAD, CRP.
